# The effect of trinitrobenzene sulfonic acid on gut-derived smooth muscle cell arachidonic acid metabolism: role of endogenous prostanoids

**DOI:** 10.1080/09629359791749

**Published:** 1997-06

**Authors:** W. E. Longo, G. S. Smith, Y. Deshpande, C. Reickenberg, D. L. Kaminski

**Affiliations:** 1 Department of Surgery Theodore Cooper Surgical Research Unit St. Louis University School of Medicine and Health Sciences Center St. Luois MO USA

## Abstract

The contribution of smooth muscle cells as a potential source of eicosanoid production during inflammatory states remains to be elucidated. We investigated the effect of trinitrobenzene sulfonic acid (TNB), a known pro-inflammatory agent, on jejunal smooth muscle cell eicosanoid production. Human gut-derived smooth muscle cells (HISM) were incubated with TNB for 1 hour. Additionally, some cells were preincubated with either dimethylthiourea, or indomethacin for 1 hour before exposure to identical concentrations of TNB. Incubation with TNB led to significant increases in PGE_2_ and 6-keto PGF-1_α_ release, but not leukotriene B_4_ release; responses which were both inhibited by dimethylthiourea and indomethacin treatment. Our results suggest that gutderived smooth muscle cells may represent an important source of proinflammatory prostanoids but not leukotrienes during inflammatory states of the intestine. The inhibition of prostanoid activity by thiourea may be mediated by suppression of cyclooxygenase activity in this cell line.


Mediators of Inflammation, 6, 237–240 (1997)

### The effect of trinitrobenzene sulfonic acid on gut-derived smooth muscle cell arachidonic acid metabolism: role of endogenous prostanoids

W. E. Longo, G. S. Smith, Y. Deshpande, C. Reickenberg and D. L. Kaminski[CA]

Department of Surgery, Theodore Cooper Surgical Research Unit, St. Louis University School of Medicine and Health Sciences Center, St. Luois, MO, USA

[CA]Corresponding author
Tel: (+1) 314 577 8372
Fax: (+1) 314 771 1945

**The contribution of smooth muscle cells as a potential source of eicosanoid production during inflammatory states remains to be elucidated. We investigated the effect of trinitrobenzene sulfonic acid (TNB), a known pro-inflammatory agent, on jejunal smooth muscle cell eicosanoid production. Human gut-derived smooth muscle cells (HISM) were incubated with TNB for 1 hour. Additionally, some cells were preincubated with either dimethylthiourea, or indomethacin for 1 hour before exposure to identical concentrations of TNB. Incubation with TNB led to significant increases in $PGE_2$ and 6-keto $PGF\text{-}1_\alpha$ release, but not leukotriene $B_4$ release; responses which were both inhibited by dimethylthiourea and indomethacin treatment. Our results suggest that gut-derived smooth muscle cells may represent an important source of proinflammatory prostanoids but not leukotrienes during inflammatory states of the intestine. The inhibition of prostanoid activity by thiourea may be mediated by suppression of cyclooxygenase activity in this cell line.**

**Key words:** Arachidonic acid, Smooth muscle, Trinitrobenzene sulfonic acid

### Introduction

Inflammatory bowel diseases, including Crohn's disease and ulcerative colitis, are multifactorial disorders whose etiology remains poorly understood. They are characterized by either mucosal or transmural necrosis that produces symptoms of crampy abdominal pain, diarrhea, and failure to thrive. A number of mediators of inflammation such as reactive oxygen metabolites, neutrophils, autacoids, cell adhesion molecules, and cytokines have been implicated as contributors to the cellular events leading to intestinal inflammation. In both experimental models of colitis and in tissue and rectal dialysates from patients with colitis, elevated levels of mucosal prostanoids, leukotrienes and cytokines have been observed.[1] With the premis that these mediators of inflammation may perpetuate illness, much effort has been directed towards the amelioration of these responses through the application and use of cyclooxygenase inhibitors and cytokine antagonists.

The effects of intestinal inflammation on gastrointestinal motility in terms of the production and effect of prostaglandins, thromboxanes, and leukotrienes has prompted a search for their physiologic role in these processes. The effects of eicosanoids on gastrointestinal motility has been studied both *in vitro* using isolated segments of esophagus, stomach, ileum and colon and *in vivo* following intravenous infusion into animals and man. There is ample evidence that eicosanoids may be involved in complex interactions that govern the control of contraction of gastrointestinal smooth muscle, as well as the promotion of inflammatory responses. It is important to point out, however, that their effects show considerable variability depending on the type of eicosanoid, the dose, the species studied and the muscle layer which is used.[2] Certainly, in patients with intestinal inflammatory disorders both diarrhea and ileus are present. The role of prostanoids as potential contributors to these responses remains to be further elucidated.

Reactive oxygen intermediates or oxygen derived free radicals such as superoxide and hydroxy radicals have been shown to induce cyclooxygenase (COX) activity. Thiourea compounds (i.e. DMTU), which are free radical scavengers, have been shown to exhibit potent gastroprotective properties against a variety of luminal insults, suggesting that they may possess anti-inflammatory properties.[3,4] To date,

© 1997 Rapid Science Publishers however, their effects on eicosanoid production during intestinal inflammatory states have not been thoroughly delineated, although there has been some suggestion that they modulate prostanoid production under both *in vivo* and *in vitro* conditions.[3–6]

Cell cultures from isolated smooth muscle cells have become invaluable for the study of fundamental physiologic and biochemical properties of muscle. Most studies have focused on contractile properties of smooth muscle cells in culture, while their role as potential contributors to eicosanoid production has been underscored. Previous studies have documented that colitis can be induced by the administration of an enema containing the contact sensitizing allergen TNB in ethanol; these chemicals have also been utilized in rabbits to produce ileitis. The present study was undertaken to investigate the effect of TNB on release of prostanoids and leukotrienes by a human small intestinal smooth muscle cell line and the subsequent impact of dimethylthiourea on TNB-induced eicosanoid release.

### Methods

Human intestinal smooth muscle cells (HISM, catalogue # 1692-CRL) were obtained from the American Type Culture Collection (Rockville, MD). Cells were maintained at 37°C in an atmosphere of 5% $CO_2$ and 100% relative humidity. Cells were split at a ratio of 1:2 upon reaching confluency. Cells were detached using 0.5 g porcine trypsin and 0.2 g tetrasodium EDTA/1 Hank's balanced salt solution (Sigma Chemical, St. Louis, MO) and then plated into 24-well plates, for experiments, or into 175-$cm^2$ flasks (Costar, Cambridge, MA) for propogation. Media was changed every 5–7 days; Dulbecco's modified Eagle's medium with 10% fetal bovine serum, 100 units/ml penicillin, 100 µg/ml streptomycin and 0·25 µg/ml amphotercin B (Sigma) was used. The cells were plated at a seeding density of $2 \times 10^5$ cells/well and allowed to grow to confluence. Viability of the cells was verified by trypan blue exclusion and morphology was evaluated by phase contrast microscopy.

Stock solutions of TNB (1 mM; Sigmal Chemicals, St. Louis, MO) and dimethythiourea (DMTU, 5 mM; Sigma Chemicals, St. Louis, MO) were diluted in Krebs–Ringer's bicarbonate. Indomethacin (Indo, 5 mM; Sigma Chemicals. St. Louis, MO) was dissolved in 5 mg/ml $Na_2CO_3$ in physiological saline. Cells were preincubated with Indo (40 µM) or DMTU (50 µM) for 1 hour in serum free media. Control cells were exposed to serum free media alone. All cells were then incubated with TNB (10 µM) for an additional 1 hour period. At the conclusion of the experiments, the cells and buffer were collected and frozen at −80°C until use.

$PGE_2$, 6 keto-$PGF1_\alpha$ and $LTB_4$ assays were performed in duplicate without separation by a competitive enzyme assay which uses an acetylcholinesterase tracer (Cayman, Ann Arbor, MI). The eicosanoid concentrations were determined by spectrophotometric analysis after addition of Ellman's reagent and compared to standard curves generated under identical conditions.

Total protein concentration was estimated colorometrically with BCA protein assay kits (Pierce, Rockford, IL). Briefly, the plates were thawed and cells incubated and permeabilized in 0·3 ml of 1 N NaOH. All samples were then incubated at 37°C for 30 min and 50 µl aliquots of blank, BSA standard or sample were mixed with 200 µl of test reagent and loaded into 96-well plates. After a 2 hour incubation period at 37°C, all samples were read in a spectrophotometric plate reader at a wavelength of 595 nm.

The concentrations of eicosanoids were expressed as pg/mg total protein. The data is presented as mean ± SEM. Statistical analysis was performed by analysis of variance. Differences between groups was determined by the least significant difference. 'Significant' indicates $P < 0{\cdot}05$.

### Results

Incubation of HISM cells with TNB produced significant and dose-dependent increases in the release of $PGE_2$ and 6-$KPGF1_\alpha$ (the stable metabolite of prostacyclin). TNB administration in the concentrations and time intervals used in this study did not significantly change $LTB_4$ production by these cells. Similarly, preincubation of HISM cells with DMTU or indomethacin had no effect on $LTB_4$ levels.

Preincubation of these cells with either indomethacin or DMTU significantly decreased both basal and TNB stimulated $PGE_2$ and 6-$KPGF1_\alpha$ release, suggesting that DMTU, like indomethacin, may have a direct inhibitory effect on smooth muscle prostaglandin release (Table 1).

### Discussion

Arachidonic acid metabolites play pivotal, but complicated and often contradictory, roles in a wide range of normal autocrine and paracrine

**Table 1**. Effect of TNB, indomethacin and DMTU on $PGE_2$, 6-KPGF1$_\alpha$, and $LTB_4$ release from HISM cells

| | $PGE_2$ | 6KPGF | $LTB_4$ |
|---|---|---|---|
| Buffer | 1042.3 ± 321.8 | 531.7 ± 125.6 | 6.7 ± 1.1 |
| Indo | 212.4 ± 42.5[a] | 113.4 ± 39.2[a] | 4.9 ± 1.3 |
| DMTU | 387.4 ± 121.3[a] | 272.2 ± 86.0[a] | 5.7 ± 1.4 |
| TNB | 1878.9 ± 324.8[a] | 1198.3 ± 335.6[a] | 4.9 ± 2.2 |
| TNB + indo | 422.4 ± 111.6[b] | 288.1 ± 79.9[b] | 5.7 ± 0.9 |
| TNB + DMTU | 597.8 ± 132.6[b] | 435.1 ± 99.2[b] | 6.6 ± 1.8 |

[a] = $P < 0.05$ from buffer.
[b] = $P < 0.05$ from TNB.

cellular interactions. The role of these mediators in intestinal inflammation has been studied both *in vivo* and *in vitro* conditions in which increased levels of arachidonic acid metabolites have been demonstrated. In a previous study $PGE_2$ and prostacyclin release were noted when CaCO2 cells were exposed to the *Clostridium difficile* toxin, a known pro-inflammatory agent.[7] Interestingly, pretreatment of these cells with indomethacin, a commonly employed cyclooxygenase inhibitor before exposure to this toxin resulted in an inhibitory effect on $PGE_2$ and 6-KPGF1$_\alpha$ but a paradoxical increase in $LTB_4$ release. Further investigations using a similar model of TNB-induced stimulation demonstrated increased release of $PGE_2$ and 6-KPGF1$_\alpha$ which correlated with elevated release of lactate dehydrogenase, confirming significant cellular injury under these conditions. Indomethacin pretreatment attenuated markedly the elevations in prostanoid release.[8]

Free radicals such as the superoxide anion, the hydroxy radical and nitric oxide are generated at sites of inflammation. These highly reactive chemical species have been shown to induce COX-2 activity. Inhibitors of reactive oxygen formation and the scavengers of reactive oxygen species have been reported to block prostanoid production by quenching the generation of hydroperoxides, which are activators of COX.[9] In rat mesangial cells, following inflammatory stimuli or growth factors, thioureas inhibited the increase in *COX-2* mRNA, COX-2 protein and $PGE_2$ synthesis.[10] This proposed mechanism of transcription of the *COX-2* gene may operate through the NFkB transcription factor.[11] The relationship between nitric oxide and COX is unclear. Nitric oxide, another free radical associated with inflammation, has been demonstrated to potentiate prostaglandin output in endothelial cells by activation of COX.[12] On the other hand, inhibition of prostaglandin release by nitric oxide has been observed in macrophages.[13]

Prostanoids act locally in a paracrine and autocrine manner and modulate cell and tissue responses in physiological and pathological states. Most whole organ models of intestinal inflammation focus on the role of epithelial cells as a major potential source of pro-inflammatory prostanoids, whereas a contributory role for enhanced eicosanoid synthesis by the underlying smooth muscle cell is often overlooked. Intestinal smooth muscle cells produce eicosanoids as well and serve as a potential source for these important inflammatory mediators. Our data demonstrates that: intestinal smooth muscle cells represent a potential source of pro-inflammatory prostanoids detected in this model of intestinal inflammation; and that thioureas such as DMTU inhibit the release of these endogeneous smooth muscle prostanoids in a manner similar to the activity of another cyclooxygenase inhibitor – indomethacin. Based upon the effect of DMTU on eicosanoid production, a novel suggestion from the present study is that thioureas may provide a potential new clinical modality for the treatment of intestinal inflammatory states.

This study suggests that human smooth muscle cells produce oxygen free radicals in response to TNB and the effect of the reactive oxygen species is attentuated by the antioxidant. Alternatively, dimethylthiourea may inhibit the prostanoid formation through a mechanism independent of the neutralization of oxygen free radicals. Further studies are underway to attempt to answer this question.

### References

1. Nassif A, Longo WE, Mazuski JE, Vernava AM, Kaminski DL. The Role of Cytokines and Platelet-Activating Factor in Inflammatory Bowel Disease: Implications for Therapy. *Dis Colon Rectum* 1996; **39**: 217–223.
2. Thor P, Konturek JW, Konturek SJ, Anderson JH. Role of prostaglandins in control of intestinal motility. *Am J Physiolol* 1985; **248**: G353–359.
3. Bulkey GB. The role of oxygen free radicals in human disease processes. *Surgery* 1983; **94**: 407–411.
4. Pihan G, Regillo C, Szabo AS. Free radicals and lipid peroxidation in ethanol or aspirin induced gastric mucosal injury. *Dig Dis Sci* 1987; **32**: 1395–1401.
5. Smith GS, Barreto JC, Schmidt KL, Tornwall MS, Miller TA. Protective Effect of Dimethylthiourea Against Mucosal Injury in Rat Stomach. Implications for Hydroxyl Radical Mechanism. *Dig Dis Sci* 1992; **37**: 1345–1355.
6. Kaufman RP, Klausner JM, Anner H, *et al.* Inhibition of Thromboxane (Tx) Synthesis by Free Radical Scavengers. *J Trauma* 1988; **28**: 458–464.
7. Stratton MD, Chandel B, Deshpande Y, *et al.* The Effect of *Clostridium difficile* Toxin on Colonocyte Prostanoid Activity. *Prostaglandins* 1994; **48**: 367–375.
8. Stratton MD, Sexe R, Peterson B, Kaminski DL, Li AP, Longo WE. The Effects of Trinitrobenzene (TNB) on Colonocyte Arachidonic Acid Metabolism. *J Surg Res* 1996; **60**: 375–378.
9. Lands WEM, Kulmacy RJ, Marshall PJ. Lipid peroxide actions in the regulation of prostaglandin biosynthesis. In: Pryor WA ed. *Free Radicals in Biology*. New York: Academic Press, Inc., 1984; 39–61.
10 Feng L, Xia Y, Garcia GE, Hwand D, Wilkinson CB. Involvement of reactive oxygen intermediates in cyclooxygenase-2 expression induced by interleukin-1, tumor necrosis factor and lipolysaccharide. *J Clin Invest* 1995; **95**: 1669–1675.

11. Brennan P, O'Neill LAJ. Effects of oxidants and antioxidants on nuclear factor kB activation in three different cell lines: evidence against a universal hypothesis involving oxygen radicals. *Biochim Biophy Acta* 1995; **1260**: 167–175.
12. Davidige ST, Baker PN, McLaughlin MK, Roberts JM. Nitric oxide produced by endothelial cells increases production of eicosanoids through activation of prostaglandin H synthase. *Circ Res* 1995; **77**: 274–283.
13. Swierkosz TA, Mitchell JA, Warner TD, Botting RM, Vane JR. Coinduction of nitric oxide synthase and cyclooxygenase: interactions betwen nitric oxide and prostanoids. *Br J Pharmacol* 1995; **114**: 1335–1342.

ACKNOWLEDGEMENT. This work was supported by USPHS Grant DK 27695.

**Received 25 March 1997; accepted 1 April 1997**
